# Development and external validation of a nomogram to predict prolonged postoperative mechanical ventilation in patients with acute type A aortic dissection

**DOI:** 10.3389/fcvm.2026.1830714

**Published:** 2026-07-08

**Authors:** Qi Yue, Jianhao Hu, Xin Li, Haiyuan Liu, Zhenxiao Ma, Chun Wu, Weibo Kong, Yuyong Liu

**Affiliations:** 1 Department of Cardiovascular Surgery, The First Affiliated Hospital of Anhui Medical University, Hefei, Anhui, China; 2 Department of Cardiac Surgery, National Cardiovascular Disease Regional Center for Anhui, The First Affiliated Hospital of Anhui Medical University, Hefei, Anhui, China; 3 Department of Cardiac Surgery Center, Beijing Anzhen Hospital, Capital Medical University, Beijing, China; 4 Department of Anesthesiology, The First Affiliated Hospital of USTC, Division of Life Sciences and Medicine, University of Science and Technology of China, Hefei, Anhui, China

**Keywords:** acute type A aortic dissection (ATAAD), least absolute shrinkage and selection operator (LASSO), nomogram, prediction model, prolonged mechanical ventilation (PMV)

## Abstract

**Background:**

Prolonged mechanical ventilation (PMV) significantly affects outcomes in patients undergoing acute type A aortic dissection (ATAAD) surgery. This study aimed to develop a nomogram to predict the risk of PMV (defined as mechanical ventilation >48 h) to help clinicians identify high-risk patients and improve outcomes.

**Methods:**

This retrospective study, designed and reported in accordance with the Transparent Reporting of a multivariable prediction model for Individual Prognosis Or Diagnosis (TRIPOD) statement, included 479 ATAAD patients from the First Affiliated Hospital of Anhui Medical University (training set) and 120 patients from Beijing Anzhen Hospital of Capital Medical University (validation set). Potential predictors were selected using Least Absolute Shrinkage and Selection Operator (LASSO) regression and multivariate logistic regression. A nomogram was then developed using the retained predictors. Its performance was evaluated using receiver operating characteristic curves, calibration curves, decision curve analysis, and clinical impact curves.

**Results:**

Ten predictors were retained in the final model: age [odds ratio (OR) 1.036, 95% confidence interval (CI) 1.016–1.058, *P* < 0.001], preoperative serum albumin (OR 0.942, 95% CI 0.903–0.984, *P* < 0.01), fibrinogen (OR 0.777, 95% CI 0.643–0.938, *P* < 0.01), standard bicarbonate (OR 0.891, 95% CI 0.798–0.996, *P* < 0.05), red cell distribution width (OR 1.325, 95% CI 1.112–1.578, *P* < 0.01), serum creatinine (Cr) (OR 1.005, 95% CI 1.000–1.011, *P* = 0.056), uric acid (OR 1.002, 95% CI 1.000–1.004, *P* < 0.05), isolated ascending aortic replacement (OR 0.578, 95% CI 0.348–0.960, *P* < 0.05), total arch replacement with frozen elephant trunk (TAR-FET) (OR 1.999, 95% CI 1.250–3.198, *P* < 0.01), and aortic cross-clamp time (OR 1.010, 95% CI 1.003–1.017, *P* < 0.01). The nomogram demonstrated good discriminatory ability (training AUC 0.796, 95% CI 0.756–0.835; validation AUC 0.765, 95% CI 0.678–0.852), excellent calibration (Hosmer–Lemeshow test: *P* = 0.229 for the training cohort, *P* = 0.855 for the validation cohort), and good clinical applicability. At the optimal Youden index cutoff (predicted probability = 0.484 in the training cohort, 0.536 in the validation cohort), the nomogram achieved a sensitivity of 78.6%, specificity of 66.7%, positive predictive value of 74.6%, and negative predictive value of 71.4% in the training cohort and the corresponding values were 76.0%, 64.4%, 78.1%, and 61.7% in the validation cohort.

**Conclusions:**

The nomogram effectively predicts the risk of PMV after ATAAD surgery, providing valuable insights for perioperative management and potentially improving patient outcomes.

## 1 Introduction

Acute type A aortic dissection (ATAAD) is an extremely life-threatening cardiovascular disease. Epidemiologic findings indicate an annual incidence of 7.2 cases of acute aortic dissection per 100,000 individuals, and 80% of the patients die from dissection rupture without timely treatment (1, 2). Currently, surgery is an indispensable modality for the treatment of ATAAD; however, perioperative hemodynamic instability and postoperative cardiac, renal, cerebral, pulmonary, and other vital organ complications have a fatal impact on patient outcomes, with respiratory complications resulting from the systemic inflammatory response being particularly significant (3–5). Prolonged mechanical ventilation (PMV) in patients is associated with longer ICU and hospital stays, increased postoperative mortality, and is often indicative of a poor prognosis. The duration of mechanical ventilation has been included by the Society of Thoracic Surgeons (STS) as one of the indicators for assessing postoperative severity in patients with ATAAD (6, 7). Therefore, we need to predict whether PMV occurs in patients after surgery based on relevant risk factors at an early stage to guide timely preventive and therapeutic interventions and improve clinical outcomes (8, 9).

Several recent studies have used preoperative and/or intraoperative risk factors to predict postoperative PMV in patients; however, these studies were all conducted at a single center and lacked external validation. In this study, we developed a predictive nomogram using data from ATAAD patients treated at the First Affiliated Hospital of Anhui Medical University and externally validated the model using an independent cohort from Beijing Anzhen Hospital, Capital Medical University. This nomogram enables individualized risk prediction of postoperative PMV and may help clinicians in tailoring perioperative management to improve patient outcomes.

## 2 Materials and methods

### 2.1 Patients

This retrospective clinical study included patients who underwent surgery for ATAAD at the Department of Cardiac Surgery of the First Affiliated Hospital of Anhui Medical University from 1 February 2019 to 31 October 2023 as the training set and patients who underwent ATAAD surgery at the Anzhen Hospital of Capital Medical University in Beijing from 1 December 2023 to 31 January 2025 as the validation set.

**Figure 1:**
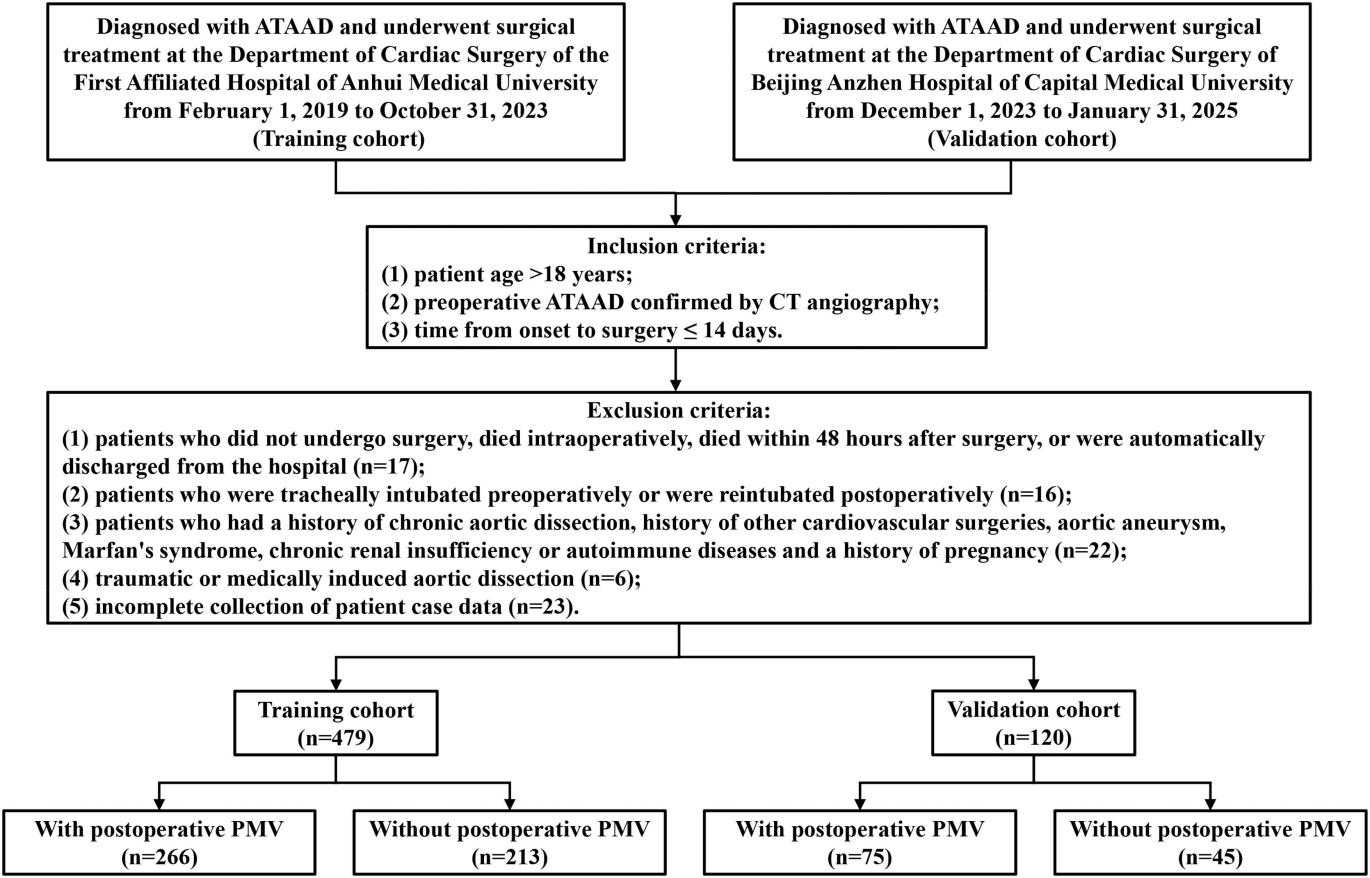
Research flowchart for this study.

Inclusion criteria for this study were (1) age >18 years, (2) preoperative ATAAD confirmed by CT angiography, and (3) time from onset to surgery ≤ 14 days. Exclusion criteria were (1) patients who did not undergo surgery, died intraoperatively, died within 48 h after surgery, or were automatically discharged from the hospital; (2) patients who were tracheally intubated preoperatively or were reintubated postoperatively; (3) patients who had a history of chronic aortic dissection, history of other cardiovascular surgeries, aortic aneurysm, Marfan's syndrome, chronic renal insufficiency or autoimmune diseases and a history of pregnancy; (4) traumatic or iatrogenic aortic dissection; and (5) incomplete patient case data. Based on the above inclusion and exclusion criteria, a total of 479 patients were included in the training set and 120 in the validation set (Figure 1). All ATAAD patients in this cohort underwent some form of ascending aortic intervention. The recorded surgical-strategy variables—isolated ascending aortic replacement (AAR), aortic root replacement, partial arch replacement, and total arch replacement with frozen elephant trunk (TAR-FET)—are not mutually exclusive and describe distinct technical components rather than the entire operation.

### 2.2 Data collection and variables

Postoperative mechanical ventilation lasting longer than 48 h was defined as PMV. The 48-h threshold was selected because it is the cutoff most commonly used in dedicated PMV studies of ATAAD and other complex aortic procedures (6, 7, 10), in which baseline ventilation times are intrinsically longer than after isolated coronary or valve surgery; a 24-h STS-based threshold would classify the majority of ATAAD patients as having PMV and reduce the prognostic discrimination of the variable. In this study, we conducted a detailed statistical analysis of variables that may cause postoperative PMV in ATAAD patients, including preoperative variables and intraoperative variables. All variables are listed in Supplementary Table 1.

The following abbreviations are used throughout the article: preoperative serum albumin (ALB), preoperative coagulation fibrinogen (FBG), preoperative blood gas standard bicarbonate (SBC), preoperative red cell distribution width (RDW), preoperative serum creatinine (Cr), preoperative uric acid (UA), aortic cross-clamp time (AOT), isolated AAR, and TAR-FET.

### 2.3 Statistical analysis

The study was designed and reported in accordance with the Transparent Reporting of a multivariable prediction model for Individual Prognosis Or Diagnosis (TRIPOD) statement (11). A completed TRIPOD checklist is provided as Supplementary File 1. The patients in this study were divided into a training set and a validation set, with external data used as the validation set. To avoid spurious effects from imputation, variables with more than 50% missing values were excluded *a priori*; preoperative C-reactive protein (CRP, 65.6% missing in the training cohort) was excluded on this basis. A sensitivity analysis using multiple imputation by chained equations (20 imputations) confirmed that re-introducing CRP did not improve model performance [pooled odds ratio (OR) 1.003, 95% confidence interval (CI) 0.981–1.026, *P* = 0.79; ΔAUC = + 0.001]. In the training set, we first applied Least Absolute Shrinkage and Selection Operator (LASSO) regression to all variables included in the analysis; 10-fold cross-validation was used to determine the optimal *λ* in the LASSO regression model, and 14 variables were retained based on the 1-SE criterion. Then, we performed multivariate logistic regression to determine independent risk factors for PMV. These finally screened factors were used to construct a nomogram. With 266 events and 10 predictors in the final model, the events-per-variable ratio was 26.6, comfortably exceeding the TRIPOD-recommended minimum of 10. For each patient in the training set, the model probability and NomoScore were calculated, and the optimal cutoff value was determined using the Youden index. Finally, the performance of the prediction model was evaluated separately in the training and validation sets. The receiver operating characteristic (ROC) curve was used to evaluate the clinical discriminatory ability of the model. Calibration was assessed using calibration curves, the calibration slope and intercept, the Brier score, and the Hosmer–Lemeshow goodness-of-fit test. Clinical decision curve analysis (DCA) was performed to evaluate the net clinical benefit of the model for decision-making support.

All data in this study were analyzed by SPSS version 27.0 and R version 4.4.2. For numerical variables with a normal distribution, data are presented as the mean ± standard deviation, and comparisons between groups were performed using the independent-samples *t*-test. For numerical variables with skewed distributions, data are presented as the median and interquartile range, and comparisons between groups were made using the Mann–Whitney *U*-test. For categorical variables, data are presented as percentages, and comparisons were made using the chi-square test (or Fisher's exact test). All reported *P* values are two-sided, and *P* values less than 0.05 were considered statistically significant.

## 3 Results

### 3.1 Patient characteristics

Based on the established inclusion and exclusion criteria, a total of 599 patients who underwent ATAAD surgery were included in this study; of these, 479 patients from the Department of Cardiac Surgery of the First Affiliated Hospital of Anhui Medical University comprised the training set, and 120 patients from the Anzhen Hospital of Capital Medical University, Beijing, China, comprised the validation set (Supplementary Table 1). The training cohort included 266 patients (55.5%) with postoperative PMV and 213 (44.5%) patients without postoperative PMV. The validation cohort included 75 (62.5%) patients with postoperative PMV and 45 (37.5%) patients without postoperative PMV. Table 1 presents the results of the baseline analysis in the training set.

**Table 1:** Baseline characteristics of patients in the training cohort.

Variables	Total (*n* = 479)	Without PMV (*n* = 213)	With PMV (*n* = 266)	Statistic	*P*
(A) Preoperative demographics, vital signs, and comorbidities					
Age (year), mean ± SD	48.68 ± 10.99	46.57 ± 11.30	50.37 ± 10.46	*t* = −3.81	<0.001
Male, *n* (%)	355 (74.11)	165 (77.46)	190 (71.43)	*χ*²=2.25	0.134
Pulse (time/min), *M* (Q1, Q3)	80.00 (76.00, 90.00)	80.00 (76.00, 88.00)	82.00 (76.00, 92.00)	*Z* = −2.10	0.035
SBP (mmHg), *M* (Q1, Q3)	129.00 (117.00, 138.00)	129.00 (119.00, 138.00)	128.50 (116.00, 139.00)	*Z* = −0.25	0.806
DBP (mmHg), *M* (Q1, Q3)	70.00 (60.00, 80.00)	71.00 (62.00, 80.00)	70.00 (60.00, 80.00)	*Z* = −1.00	0.317
Height (cm), *M* (Q1, Q3)	172.00 (166.50, 175.00)	173.00 (170.00, 176.00)	170.00 (165.00, 175.00)	*Z* = −2.66	0.008
Weight (kg), *M* (Q1, Q3)	75.00 (70.00, 85.00)	78.00 (70.00, 85.00)	75.00 (68.50, 85.00)	*Z* = −1.04	0.297
BMI (kg/m²), *M* (Q1, Q3)	26.03 (23.66, 28.31)	26.12 (23.53, 27.78)	25.95 (23.88, 28.73)	*Z* = −0.38	0.701
Time of occurrence (day), *M* (Q1, Q3)	1.00 (0.80, 3.00)	1.00 (1.00, 4.00)	1.00 (0.71, 2.00)	*Z* = −3.16	0.002
Smoking, *n* (%)	162 (33.82)	61 (28.64)	101 (37.97)	*χ*²=4.60	0.032
Hypertension, *n* (%)	265 (55.32)	96 (45.07)	169 (63.53)	*χ*²=16.31	<0.001
Diabetes mellitus, *n* (%)	19 (3.97)	6 (2.82)	13 (4.89)	*χ*²=1.33	0.249
History of cardiovascular disease, *n* (%)	32 (6.68)	12 (5.63)	20 (7.52)	*χ*²=0.67	0.412
History of aortic surgery, *n* (%)	6 (1.25)	3 (1.41)	3 (1.13)	*χ*²=0.00	1.000
History of valve surgery, *n* (%)	4 (0.84)	2 (0.94)	2 (0.75)	*χ*²=0.00	1.000
History of bypass grafting, *n* (%)	2 (0.42)	1 (0.47)	1 (0.38)	-	1.000
Coronary artery disease, *n* (%)	10 (2.09)	4 (1.88)	6 (2.26)	*χ*²=0.00	1.000
Numbness of the limbs, *n* (%)	33 (6.89)	14 (6.57)	19 (7.14)	*χ*²=0.06	0.807
Respiratory disease, *n* (%)	42 (8.77)	19 (8.92)	23 (8.65)	*χ*²=0.01	0.916
(B) Preoperative laboratory measurements					
WBC (10⁹/L), *M* (Q1, Q3)	9.31 (7.18, 12.59)	9.42 (7.24, 12.53)	9.28 (7.07, 12.67)	*Z* = −0.08	0.936
PLT (10⁹/L), *M* (Q1, Q3)	94.00 (57.00, 151.00)	115.00 (78.00, 170.00)	77.00 (47.20, 120.25)	*Z* = −6.25	<0.001
RBC (10¹²/μL), *M* (Q1, Q3)	3.44 (2.95, 4.04)	3.55 (3.16, 4.22)	3.31 (2.85, 3.84)	*Z* = −4.20	<0.001
Hb (g/L), mean ± SD	110.65 ± 23.75	114.90 ± 22.83	107.25 ± 23.97	*t* = 3.55	<0.001
Neu (10⁹/L), *M* (Q1, Q3)	7.80 (5.64, 10.32)	7.74 (5.67, 10.55)	7.83 (5.61, 10.29)	*Z* = −0.21	0.833
RDW (%), *M* (Q1, Q3)	13.50 (12.70, 14.30)	13.00 (12.50, 13.80)	13.80 (13.10, 14.50)	*Z* = −6.35	<0.001
BUN (mmol/L), *M* (Q1, Q3)	9.30 (7.30, 11.70)	8.40 (6.40, 10.50)	10.10 (8.20, 12.40)	*Z* = −5.98	<0.001
Cr (μmol/L), *M* (Q1, Q3)	104.50 (78.50, 134.50)	93.50 (71.00, 117.30)	112.95 (86.15, 152.10)	*Z* = −5.45	<0.001
UA (μmol/L), *M* (Q1, Q3)	334.40 (247.15, 431.15)	305.40 (231.60, 406.70)	354.85 (266.07, 454.35)	*Z* = −3.99	<0.001
ALT (U/L), *M* (Q1, Q3)	20.00 (14.00, 36.00)	20.00 (14.00, 32.00)	20.00 (15.00, 40.00)	*Z* = −1.22	0.222
AST (U/L), *M* (Q1, Q3)	54.00 (39.00, 79.00)	49.00 (36.00, 66.00)	61.00 (43.00, 101.00)	*Z* = −4.81	<0.001
ALB (g/L), *M* (Q1, Q3)	31.50 (28.20, 35.00)	32.90 (29.80, 36.90)	30.40 (26.90, 33.90)	*Z* = −5.40	<0.001
FBG (g/L), *M* (Q1, Q3)	2.77 (2.07, 3.44)	2.89 (2.46, 3.84)	2.42 (1.80, 3.15)	*Z* = −6.02	<0.001
INR, *M* (Q1, Q3)	1.18 (1.09, 1.31)	1.16 (1.09, 1.27)	1.19 (1.09, 1.35)	*Z* = −1.95	0.051
PCO₂ (mmHg), *M* (Q1, Q3)	33.20 (30.30, 36.85)	33.80 (30.50, 37.00)	33.00 (30.00, 36.70)	*Z* = −1.70	0.089
SBC (mmol/L), *M* (Q1, Q3)	23.59 (22.40, 24.75)	24.00 (23.10, 25.10)	23.20 (22.00, 24.60)	*Z* = −4.33	<0.001
Ejection fraction (%), *M* (Q1, Q3)	61.76 (60.00, 65.00)	61.76 (60.00, 66.00)	61.76 (60.00, 64.00)	*Z* = −1.18	0.239
(C) Intraoperative variables					
Extracorporeal circulation time (min), *M* (Q1, Q3)	205.00 (175.00, 235.00)	190.00 (160.00, 214.00)	217.50 (188.00, 246.75)	*Z* = −6.58	<0.001
Aortic cross-clamp time (min), *M* (Q1, Q3)	113.00 (93.00, 135.00)	102.00 (87.00, 124.00)	119.50 (101.00, 143.75)	*Z* = −5.62	<0.001
Surgical time (h), *M* (Q1, Q3)	7.94 (7.00, 8.50)	7.94 (6.75, 7.94)	7.94 (7.19, 9.00)	*Z* = −4.86	<0.001
Isolated ascending aortic replacement (AAR), *n* (%)	115 (24.01)	65 (30.52)	50 (18.80)	*χ*²=8.90	0.003
Aortic root replacement, *n* (%)	194 (40.50)	87 (40.85)	107 (40.23)	*χ*²=0.02	0.891
Wheat's procedure, *n* (%)	2 (0.42)	1 (0.47)	1 (0.38)	-	1.000
DAVID procedure, *n* (%)	1 (0.21)	1 (0.47)	0 (0.00)	-	0.445
Partial arch replacement, *n* (%)	27 (5.64)	11 (5.16)	16 (6.02)	*χ*²=0.16	0.688
Total arch replacement with FET (TAR-FET), *n* (%)	337 (70.35)	123 (57.75)	214 (80.45)	*χ*²=29.24	<0.001
CABG, *n* (%)	30 (6.26)	11 (5.16)	19 (7.14)	*χ*²=0.79	0.374

*t*, *t*-test; *Z*, Mann–Whitney test; *χ*², chi-square test, -; Fisher's exact test. SD, standard deviation; *M*, median; Q1, first quartile; Q3, third quartile. Surgical-strategy variables (AAR, aortic root replacement, partial arch replacement, TAR-FET) are not mutually exclusive; every patient underwent at least some form of ascending-aortic intervention. Preoperative CRP was excluded due to >50% missing values.

BMI, body mass index; FET, frozen elephant trunk; WBC, white blood cell count; PLT, platelet count; RBC, red blood cell count; Hb, hemoglobin; Neu, neutrophil count; RDW, red blood cell distribution width; BUN, blood urea nitrogen; Cr, serum creatinine; UA, uric acid; ALB, albumin; ALT, alanine aminotransferase; AST, aspartate aminotransferase; FBG, fibrinogen; INR, international normalized ratio; SBC, standard bicarbonate; CABG, coronary artery bypass graft; DAVID, valve-sparing aortic root replacement; AAR, isolated ascending aortic replacement; TAR-FET, total arch replacement with frozen elephant trunk.

### 3.2 Determination of independent predictors for PMV

**Figure 2:**
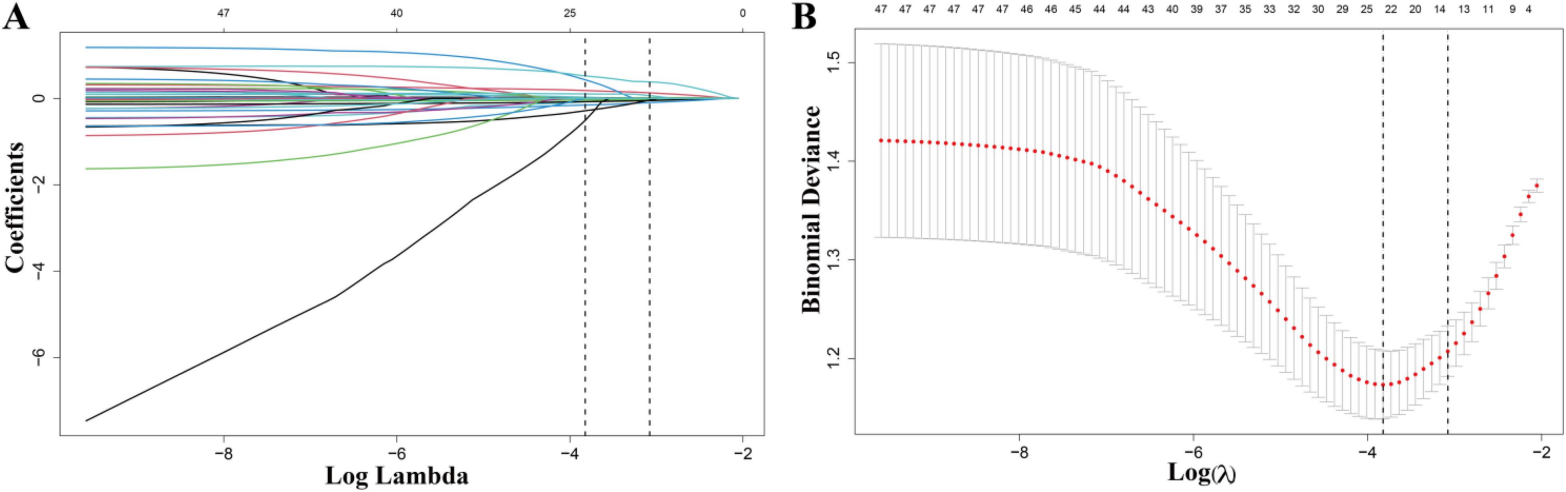
Screened variables with LASSO regression. **(A)** Plot of LASSO coefficient profiles containing 48 variables. **(B)** Ten-fold cross-validation to select the optimal *λ* in the LASSO regression model, where the value corresponding to the left vertical line is the minimum criterion and the value corresponding to the right vertical line is the 1-SE standard error. A total of 14 variables were screened by LASSO regression.

We first performed LASSO regression of preoperative and intraoperative variables that might influence postoperative PMV. Using the 1-SE criterion, 14 variables were selected: age, ALB, PLT, RDW, blood urea nitrogen, Cr, UA, FBG, SBC, extracorporeal circulation time, aortic cross-clamp time, hypertension, isolated ascending aortic replacement, and total arch replacement with FET (Figure 2). Subsequently, multivariate logistic regression with backward elimination was applied to the selected variables (Table 2). Finally, 10 predictors were retained in the final model: ALB, FBG, SBC, AAR, age, RDW, Cr, UA, AOT, and TAR-FET. Because the per-unit odds ratio for Cr was associated with a marginal *P* value (0.056), these variables are described as predictors retained in the final model rather than as strictly independent risk factors (see Discussion).

**Table 2:** Multivariate logistic regression finalizing the identification of key variables associated with postoperative PMV in patients.

Characteristics	OR	95% CI	*P*
Age (years)	1.036	1.016–1.058	<0.001
ALB (g/L)	0.942	0.903–0.984	<0.01
RDW (%)	1.325	1.112–1.578	<0.01
Cr (μmol/L)	1.005	1.000–1.011	0.056
UA (μmol/L)	1.002	1.000–1.004	<0.05
FBG (g/L)	0.777	0.643–0.938	<0.01
SBC (mmol/L)	0.891	0.798–0.996	<0.05
AOT—aortic cross-clamp time (min)	1.010	1.003–1.017	<0.01
AAR—isolated ascending aortic replacement, *n* (%)	0.578	0.348–0.960	<0.05
TAR-FET—total arch replacement with frozen elephant trunk, *n* (%)	1.999	1.250–3.198	<0.01

Age, patient age in years; ALB, serum albumin; RDW, red blood cell distribution width; Cr, serum creatinine; UA, uric acid; FBG, fibrinogen; SBC, standard bicarbonate; AOT, aortic cross-clamp time; AAR, isolated ascending aortic replacement; TAR-FET, total arch replacement with frozen elephant trunk; OR, odds ratio; CI, confidence interval.

### 3.3 Construction of a nomogram

**Figure 3:**
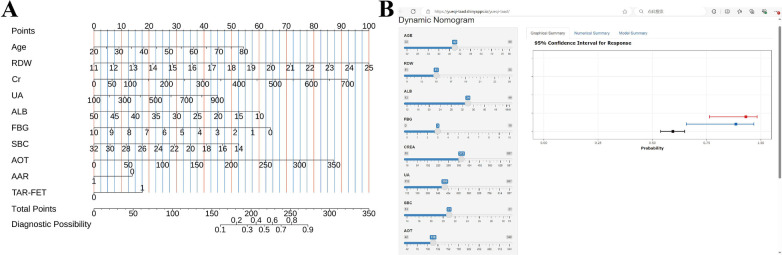
Nomogram constructed using the screened variables. **(A)** Nomogram constructed from independent predictors identified by multivariate logistic regression. AOT, aortic cross-clamp time; AAR, isolated ascending aortic replacement (0 = no, 1 = yes); TAR-FET, total arch replacement with frozen elephant trunk (0 = no, 1 = yes). **(B)** Web-based dynamic nomogram. In the web interface, “SUP” and “CREA” are displayed as the original labels for TAR-FET (total arch replacement with frozen elephant trunk) and Cr (serum creatinine), respectively.

The 10 independent risk and protective factors screened by the multivariate logistic regression model were used to construct a clinical predictive nomogram. Each predictor was assigned a score, and the total score corresponded to the probability of postoperative PMV (Figure 3A). To facilitate clinical application and enable real-time prediction, a dynamic web-based version of the nomogram was developed and is available at: https://yueqi-taad.shinyapps.io/yueqi-taad/ (Figure 3B). We calculated the model probability and NomoScore for each patient in the training and validation sets separately and then determined the optimal cutoff value. The optimal cutoff value for the model probability was 0.484 in the training cohort and 0.536 in the validation cohort, with corresponding NomoScores of 214.94 and 230.51.

### 3.4 Evaluation of clinical diagnostic predictive model

**Figure 4:**
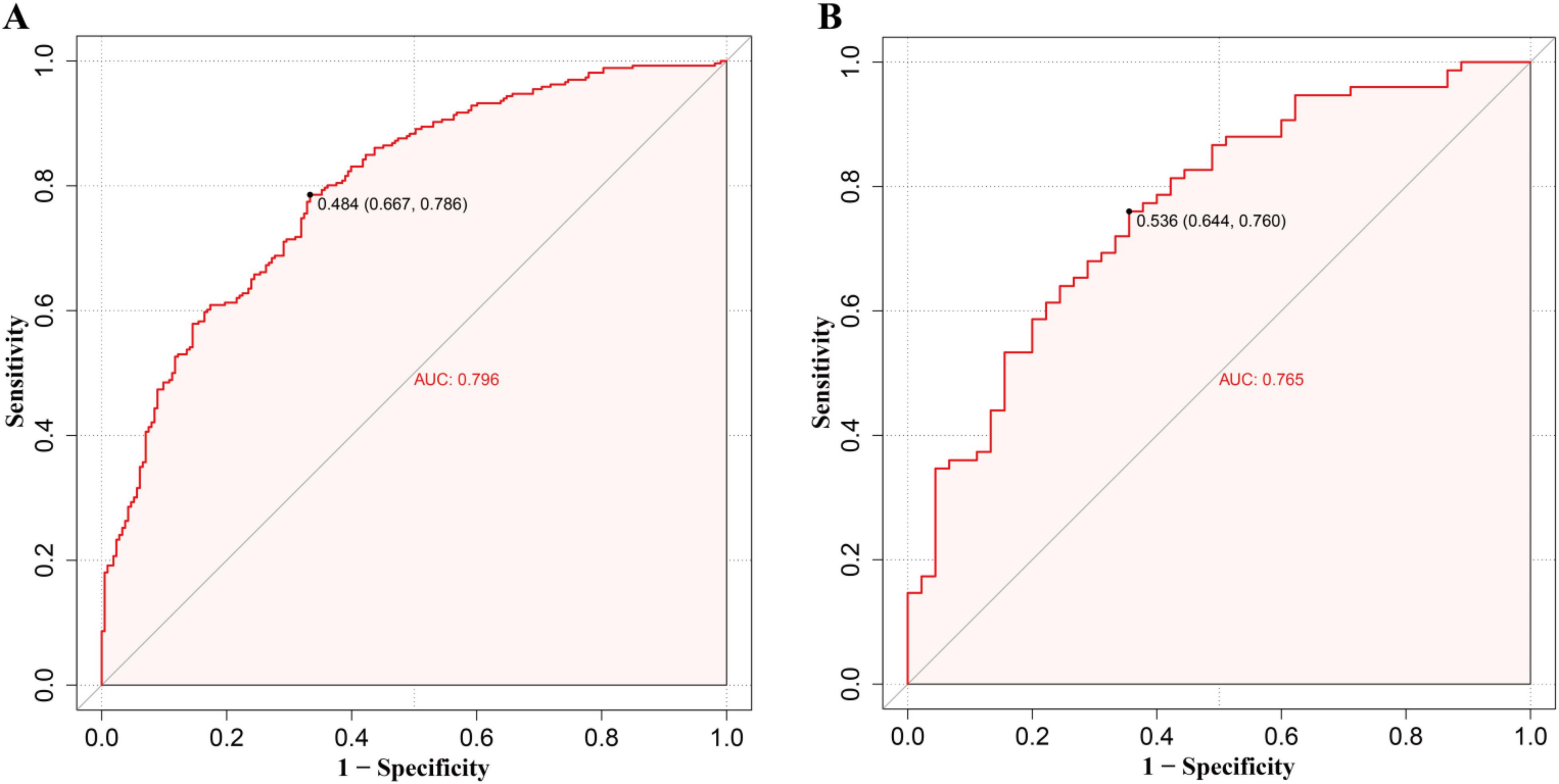
ROC curves were plotted to test model discrimination. **(A)** ROC curve in the training cohort. **(B)** ROC curve in the validation cohort.

To evaluate the discriminatory performance of the model, ROC analysis was conducted in both cohorts. The model demonstrated good discrimination, with an AUC of 0.796 (95% CI 0.756–0.835) in the training set (Figure 4A) and 0.765 (95% CI 0.678–0.852) in the validation set (Figure 4B). At the optimal Youden index cutoff, the nomogram achieved a sensitivity of 78.6%, specificity of 66.7%, positive predictive value of 74.6%, negative predictive value of 71.4%, and overall accuracy of 73.3% in the training cohort; the corresponding metrics were 76.0%, 64.4%, 78.1%, 61.7%, and 71.7% in the validation cohort (Supplementary Table 3).

**Figure 5:**
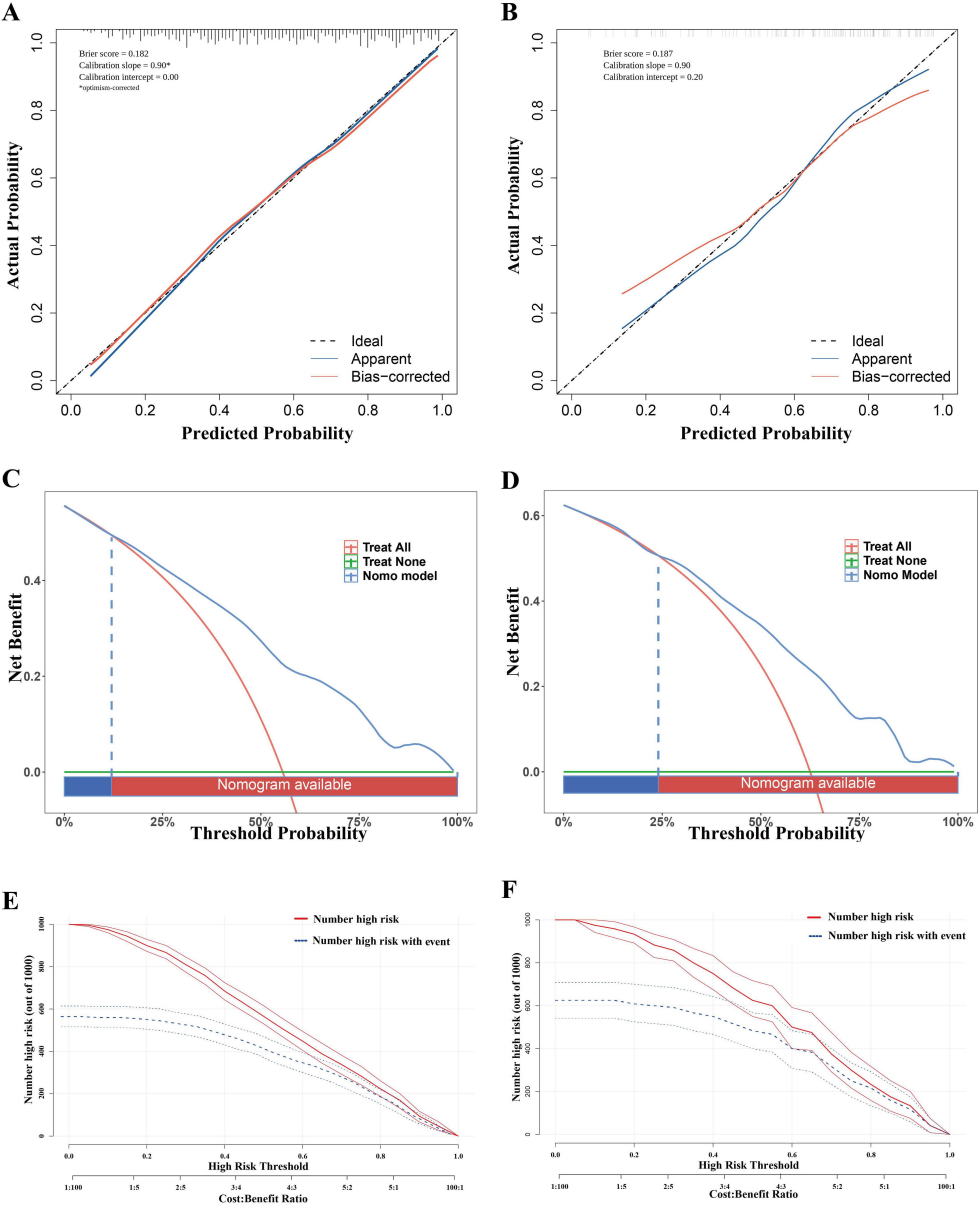
Calibration, decision, and clinical impact curves of the nomogram. **(A)** Calibration curve for the training set after 500 bootstraps. **(B)** Calibration curve for the validation set after 500 bootstraps. The Brier score, calibration slope, and calibration intercept for each cohort are annotated in **(A)** and **(B) (C)** Decision curve for the training set. **(D)** Decision curve for the validation set. **(E, F)** Clinical impact curves in the training and validation cohorts, respectively.

To assess the agreement between predicted and observed probabilities, calibration curves were plotted for both cohorts. The results showed good agreement in both the training and validation cohorts. Bootstrapping with 500 resamples also demonstrated good calibration (Figure 5). The Hosmer–Lemeshow goodness-of-fit test showed no significant deviation, with *χ*² = 10.543 (*P* = 0.229) in the training set and *χ*² = 4.022 (*P* = 0.855) in the validation set, indicating adequate model fit. Quantitative calibration metrics confirmed close agreement between predicted and observed risk: the Brier score was 0.182 in the training cohort and 0.187 in the validation cohort, while the calibration slope and intercept were 0.90 (bootstrap optimism-corrected over 500 resamples) and 0.00 in the training cohort and 0.90 and 0.20 in the validation cohort, respectively, all of which are close to their ideal values of 1 and 0 (Figures 5A,B).

To validate the clinical applicability of the model, we generated DCA plots and clinical impact curves. Both analyses indicated that the nomogram provided a favorable net clinical benefit across a range of threshold probabilities (Figure 5).

## 4 Discussion

It is well known that mechanical ventilation plays an important role in the postoperative management of patients undergoing major surgery. Mechanical ventilation can effectively support respiratory function after surgery, thereby facilitating the rapid recovery of cardiopulmonary function (12). However, prolonged mechanical ventilation is associated with inherent risks and complications that may adversely affect patient outcomes. Therefore, timely postoperative extubation is of paramount importance, and mastering the optimal timing of extubation significantly influences patient prognosis (13). PMV is a common and serious complication after cardiovascular surgery. ATAAD surgery is frequently associated with postoperative PMV because of its long operative duration and the high degree of surgical trauma (14, 15). Postoperative PMV is often accompanied by ventilator-associated pneumonia, ventilator-induced lung injury, systemic hemodynamic disturbances, systemic volume overload due to renal failure, and sepsis, which can collectively lead to longer ICU stay and, in turn, exacerbate the progression of the above complications, thereby jeopardizing the patient's life.

In summary, there is an urgent need to develop a risk prediction model for PMV that enables proactive adjustment of diagnostic and therapeutic strategies and the implementation of preventive measures to improve patient outcomes.

### 4.1 Comparison with previously published PMV prediction models in ATAAD

To our knowledge, three prior nomograms have specifically addressed PMV after ATAAD: Yu et al. (6) (*n* = 452, single-center Nanjing cohort, no external validation, AUC=0.78), Luo et al. (7) (*n* = 1,049, large single-center Nanjing cohort spanning 2011–2019, no external validation, AUC=0.79), and Xie et al. (10) (*n* = 381, restricted to the triple-branched stent-graft subgroup, AUC=0.80). The principal similarities with our work are the consistent identification of age, preoperative renal function (Cr/UA), albumin, RDW, and cardiopulmonary-bypass-related variables as predictors, supporting the convergent validity of these features across independent Chinese cohorts, and comparable discriminative performance (AUC 0.75–0.80). The principal differences and unique contributions of the present study are as follows: (a) to our knowledge, this is the only ATAAD-PMV nomogram with formal external validation across two geographically distinct centers (Hefei development cohort, Beijing validation cohort); (b) we incorporate surgical strategy variables (isolated ascending aortic replacement vs. total arch replacement with frozen elephant trunk), which are not stratified in previous models, allowing risk to be adjusted to the actual operative plan; and (c) we provide a dynamic web-based nomogram for bedside use, addressing a translational gap noted by the previous authors.

Age has been identified as a risk factor for PMV after ATAAD surgery and is also associated with increased early in-hospital mortality (16, 17). Elderly patients with many underlying diseases and poorer general health conditions, often combined with hypertension, diabetes mellitus, and cardiopulmonary insufficiency, are typically in a compromised physical condition prior to surgery. In addition, they tolerate anesthesia and surgery less well and respond poorly to medications, contributing to a systemic inflammatory state, hormonal dysregulation, and impaired immune function. These factors collectively contribute to a higher risk of postoperative PMV (18, 19). Therefore, it is extremely important to individualize the surgical plan, anesthesia dosage, and perioperative fluid management for elderly patients to help reduce the risk of postoperative PMV.

Previous studies have reported that elevated serum creatinine, as an independent risk factor, is strongly associated with early postoperative mortality and PMV in ATAAD patients (20). In our final multivariable model, the per-unit OR for Cr was 1.005 (95% CI 1.000–1.011) with a marginal *P* value of 0.056. Although this value was fractionally above the conventional *α* = 0.05, we retained Cr in the final nomogram for three reasons. First, the LASSO procedure with 10-fold cross-validation (1-SE *λ*) retained Cr, indicating that it provides non-redundant predictive information beyond the other 13 candidate variables. Second, when re-expressed per 10 μmol/L, the OR becomes 1.053, with a 95% CI that no longer crosses unity, reflecting the small per-unit effect of a continuous covariate measured in μmol/L. Third, Cr has strong biological plausibility, as renal insufficiency causes metabolic acidosis, fluid overload, and respiratory muscle weakness, all of which prolong the need for mechanical ventilation; moreover, impaired renal function has been independently associated with PMV after ATAAD in multiple prior reports (6, 7, 10). To formally test the impact of this decision, we conducted a head-to-head sensitivity analysis comparing the 10-variable model (with Cr) and a re-fitted nine-variable model (without Cr); both models were evaluated by the DeLong test for paired AUCs. The nine-variable model produced essentially identical discrimination (training AUC 0.791, 95% CI 0.751–0.831; validation AUC 0.762, 95% CI 0.672–0.851; DeLong *P* = 0.225 and 0.639, respectively) but slightly worse Brier score (0.185 vs. 0.182 in training) and required compensatory inflation of the UA coefficient (OR per μmol/L 1.0035, *P* < 0.001) to absorb the lost predictive information. The model with Cr was therefore retained as the primary nomogram, with the nine-variable alternative reported in Supplementary Table 2 for transparency. Clinically, blood creatinine, as one of the main indicators for assessing renal function, has been reported to be strongly associated with PMV after ATAAD. In this study, we also found that Cr and UA play critical roles as risk factors for postoperative PMV in ATAAD patients (21, 22). Renal insufficiency causes metabolic acidosis, which increases respiratory work and respiratory muscle fatigue, thereby leading to longer mechanical ventilation. Moreover, this study also found that SBC, an important marker of systemic acid-base balance, was associated with an elevated risk of PMV. Therefore, it is crucial to optimize preoperative renal function and initiate renal replacement therapy when necessary to reduce the risk of postoperative PMV.

RDW reflects the presence of anemia and the degree of inflammation in the body. RDW is a parameter used to assess the heterogeneity of erythrocyte size distribution. Elevated RDW is often associated with anemia, and impaired erythrocyte deformability, as well as decreased oxygen-carrying capacity, tends to lead to tissue hypoxia, which increases the risk of postoperative pulmonary complications and thus prolongs the duration of mechanical ventilation (23). In addition, elevated RDW reflects the inflammatory state of the body, and aggravation of this inflammatory state in the postoperative period leads to an increased risk of pulmonary complications (24, 25). In conclusion, high RDW is closely associated with a longer ICU stay and higher mortality in patients; therefore, timely correction of anemia and preoperative control of systemic inflammation are essential for improving patient outcomes.

The role played by coagulation in postoperative PMV should not be neglected. The decline in coagulation function of patients is related to impaired liver and kidney function, as the liver and kidneys are important organs for synthesizing certain coagulation factors. Impaired liver and kidney function directly affects the synthesis of these factors, leading to coagulation dysfunction (26). In this study, UA, Cr, ALB, and FBG can directly or indirectly reflect the preoperative coagulation status of patients. Coagulation dysfunction directly leads to decreased wound hemostasis, resulting in excessive intraoperative or postoperative bleeding, which in turn requires more prolonged mechanical ventilation to support respiration. In addition, coagulation disorders can cause abnormalities in the fibrinolytic process, resulting in unstable clots, which likewise increase the risk of bleeding and prolong the duration of mechanical ventilation; therefore, timely correction of coagulation abnormalities in the preoperative period is conducive to reducting PMV postoperatively (27, 28).

Aortic cross-clamping is essential in cardiovascular surgery, and this study found that aortic cross-clamp time is associated with postoperative PMV. First, prolonged cross-clamping is associated with ischemia–reperfusion injury, which may increase the risk of organ injury and prolong mechanical ventilation. In addition, aortic cross-clamping induces a stress response in the body, leading to the release of inflammatory mediators that may increase the risk of multiorgan failure, thus requiring longer mechanical ventilation support (29–31). The duration of aortic cross-clamping also increases the total surgical duration and may lead to hemodynamic instability, which contributes to the development of postoperative PMV.

Differences in surgical procedures also increase the risk of postoperative PMV. The study found that patients who underwent total arch replacement with FET were more likely to develop postoperative PMV than those who underwent isolated ascending aortic replacement alone. Isolated ascending aortic replacement is used for simpler and more limited aortic lesions, which are mainly confined to the ascending aorta, whereas total arch replacement with FET is a more modern and complex procedure that also treats the aortic arch, providing a broader scope of treatment and better long-term outcomes. Compared with isolated ascending aortic replacement, total arch replacement with FET is more extensive, requires a longer operative time, is associated with more postoperative complications, and is therefore more likely to result in postoperative PMV (32).

### 4.2 Relationship with the STS ascending aorta and aortic root surgery risk calculator

In April 2025, the Society of Thoracic Surgeons released a dedicated risk calculator for ascending aorta and aortic root surgery (33), derived from more than 67,000 procedures in the STS National Database. This important tool provides individualized estimates of operative mortality and composite major morbidity for four operative groups: Bentall + AVR, ascending aorta + AVR, valve-sparing root replacement, and isolated ascending aorta surgery. It is, however, complementary to, rather than a direct substitute for, our nomogram. A direct numerical comparison was not performed because the STS calculator targets different endpoints and procedural categories, for three reasons. First, the STS calculator does not provide a standalone predicted probability for PMV, which is the specific outcome targeted in this study; PMV is only one of several components of its composite morbidity endpoint. Second, the STS development cohort was drawn predominantly from Western patients undergoing largely elective ascending aorta and root surgery, whereas our cohort consists exclusively of acute, mostly arch-involving ATAAD repairs at two Chinese tertiary centers. Third, total arch replacement with frozen elephant trunk—the most common ATAAD strategy in our setting (70.4% of patients)—is not represented in the STS procedural groups. The two tools therefore address different but complementary clinical questions: the STS calculator informs decision-making regarding elective ascending aorta surgery, whereas the present nomogram is intended to anticipate respiratory recovery in ATAAD patients, for whom surgery is already mandatory. Prospective external validation of the STS calculator in Asian ATAAD cohorts is an attractive direction for future work.

Although this study adopted a multicenter design, several limitations should be noted. First, the retrospective nature of data collection across two centers may have introduced inconsistencies in the documentation of perioperative variables, particularly for less commonly recorded procedures: six of 479 (1.3%) records in the training cohort had all surgical-strategy flags coded as 0, most plausibly reflecting data-entry omissions of ancillary procedures (their recorded aortic cross-clamp and bypass times confirm that surgery was in fact performed). Second, preoperative CRP had more than 50% missing values and was therefore excluded from the analysis, so its potential role as a predictor of PMV could not be fully evaluated in the present dataset. A multiple-imputation sensitivity analysis did not identify a significant independent association between CRP and PMV after adjustment for the other 10 predictors, but prospective studies with standardized CRP collection are warranted to confirm this finding. Third, while the total sample size included multicenter data, the subgroup sample size for complex procedures (such as the WHEAT and DAVID variants) remained too small to support stratified analyses. Fourth, the definition of PMV (mechanical ventilation >48 h) may have varying clinical thresholds across institutions; the discrimination of the nomogram at alternative thresholds (24 or 72 h) should be examined in future work. In addition, patients who died within 48 h of surgery were excluded because PMV cannot, by definition, be ascertained in these patients; as they represented some of the most severely ill patients, this exclusion may introduce a degree of survivorship bias and could limit the applicability of the nomogram to the highest-risk patients, a constraint that should be borne in mind when interpreting the model. Moreover, because two of the 10 predictors—aortic cross-clamp time and the surgical strategy actually performed—become known only during or at the end of the operation, the present nomogram is intended primarily for intraoperative-to-early-postoperative risk assessment and respiratory recovery planning rather than as a purely preoperative counseling tool; nonetheless, its preoperative laboratory and demographic predictors can support provisional preoperative risk estimation, and the development of a dedicated preoperative-only model is a worthwhile direction for future work. Finally, the study identified associations between risk factors and PMV but did not clarify the underlying mechanisms or pathophysiological pathways, necessitating further validation through large-scale multicenter studies and mechanistic explorations.

## 5 Conclusion

In this study, a nomogram was constructed based on pre- and intraoperative risk factors, and validation analyses demonstrated its excellent discriminatory ability, calibration, and clinical applicability. The establishment of this diagnostic prediction model enables personalized risk stratification for ATAAD patients, facilitating proactive optimization of perioperative management to improve clinical outcomes. This evidence-based tool has significant clinical value for enhancing the precision of care and the overall prognosis of this high-risk patient population.

## Data Availability

The datasets presented in this article are not readily available because the datasets generated and/or analyzed during the current study are not publicly available due to privacy concerns of the research committee but are available from the corresponding author upon reasonable request. Requests to access the datasets should be directed to YL, yfy1132839@fy.ahmu.edu.cn.
