# Supplementary material for: Development and external validation of a nomogram to predict prolonged postoperative mechanical ventilation in patients with acute type A aortic dissection
Source: Front Cardiovasc Med. 2026 Jul 8;13:1830714. doi: 10.3389/fcvm.2026.1830714 (PMC13388465; doi:10.3389/fcvm.2026.1830714)
Supplement: Supplementary file 1 [file Datasheet1.docx]

**Supplementary Table 1. Baseline characteristics of patients in the training and validation cohorts (n = 599).**

| **Variables** | **Total (n = 599)** | **Training (n = 479)** | **Validation (n = 120)** | **Statistic** | ***P*** |
| --- | --- | --- | --- | --- | --- |
| ***A. Preoperative demographics, vital signs and comorbidities*** | | | | | |
| Age (year), Mean ± SD | 48.28 ± 11.13 | 48.68 ± 10.99 | 46.70 ± 11.56 | t=1.75 | 0.081 |
| Male, n (%) | 450 (75.13) | 355 (74.11) | 95 (79.17) | χ²=1.31 | 0.252 |
| Pulse (time/min), M (Q1, Q3) | 80.00 (76.00, 90.00) | 80.00 (76.00, 90.00) | 82.00 (76.00, 98.00) | Z=-0.88 | 0.382 |
| SBP (mmHg), M (Q1, Q3) | 128.00 (117.00, 138.00) | 129.00 (117.00, 138.00) | 125.00 (116.00, 135.00) | Z=-1.07 | 0.285 |
| DBP (mmHg), M (Q1, Q3) | 71.00 (60.00, 80.00) | 70.00 (60.00, 80.00) | 71.00 (60.00, 80.00) | Z=-0.01 | 0.991 |
| Height (cm), M (Q1, Q3) | 172.00 (166.00, 175.00) | 172.00 (166.50, 175.00) | 172.00 (165.00, 175.00) | Z=-0.69 | 0.493 |
| Weight (kg), M (Q1, Q3) | 75.00 (70.00, 85.00) | 75.00 (70.00, 85.00) | 75.00 (67.25, 81.50) | Z=-0.80 | 0.421 |
| BMI (kg/m²), M (Q1, Q3) | 25.95 (23.64, 28.04) | 26.03 (23.66, 28.31) | 25.68 (23.44, 27.69) | Z=-1.02 | 0.310 |
| Time of occurrence (day), M (Q1, Q3) | 1.00 (0.83, 3.00) | 1.00 (0.80, 3.00) | 1.00 (1.00, 3.00) | Z=-1.23 | 0.219 |
| Smoking, n (%) | 216 (36.06) | 162 (33.82) | 54 (45.00) | χ²=5.20 | 0.023 |
| Hypertension, n (%) | 335 (55.93) | 265 (55.32) | 70 (58.33) | χ²=0.35 | 0.553 |
| Diabetes mellitus, n (%) | 21 (3.51) | 19 (3.97) | 2 (1.67) | χ²=0.90 | 0.343 |
| History of cardiovascular disease, n (%) | 38 (6.34) | 32 (6.68) | 6 (5.00) | χ²=0.46 | 0.499 |
| History of aortic surgery, n (%) | 9 (1.50) | 6 (1.25) | 3 (2.50) | χ²=0.34 | 0.559 |
| History of valve surgery, n (%) | 5 (0.83) | 4 (0.84) | 1 (0.83) | χ²=0.00 | 1.000 |
| History of bypass grafting, n (%) | 2 (0.33) | 2 (0.42) | 0 (0.00) | - | 1.000 |
| Coronary artery disease, n (%) | 11 (1.84) | 10 (2.09) | 1 (0.83) | χ²=0.29 | 0.593 |
| Numbness of the limbs, n (%) | 42 (7.01) | 33 (6.89) | 9 (7.50) | χ²=0.05 | 0.815 |
| Respiratory disease, n (%) | 55 (9.18) | 42 (8.77) | 13 (10.83) | χ²=0.49 | 0.484 |
| ***B. Preoperative laboratory measurements*** | | | | | |
| WBC (10⁹/L), M (Q1, Q3) | 9.25 (7.07, 12.43) | 9.31 (7.18, 12.59) | 8.85 (6.61, 11.78) | Z=-1.95 | 0.052 |
| PLT (10⁹/L), M (Q1, Q3) | 93.00 (57.00, 151.00) | 94.00 (57.00, 151.00) | 81.50 (55.00, 136.00) | Z=-0.98 | 0.326 |
| RBC (10¹²/μL), M (Q1, Q3) | 3.43 (2.95, 4.04) | 3.44 (2.95, 4.04) | 3.38 (2.98, 3.97) | Z=-0.06 | 0.954 |
| Hb (g/L), Mean ± SD | 110.46 ± 23.42 | 110.65 ± 23.75 | 109.68 ± 22.12 | t=0.41 | 0.684 |
| Neu (10⁹/L), M (Q1, Q3) | 7.74 (5.57, 10.04) | 7.80 (5.64, 10.32) | 7.05 (4.97, 9.24) | Z=-2.14 | 0.033 |
| RDW (%), M (Q1, Q3) | 13.50 (12.80, 14.40) | 13.50 (12.70, 14.30) | 13.50 (12.80, 14.60) | Z=-0.55 | 0.581 |
| BUN (mmol/L), M (Q1, Q3) | 9.40 (7.30, 11.75) | 9.30 (7.30, 11.70) | 9.40 (7.30, 12.10) | Z=-0.07 | 0.943 |
| Cr (μmol/L), M (Q1, Q3) | 104.90 (78.95, 137.70) | 104.50 (78.50, 134.50) | 106.50 (81.62, 142.10) | Z=-0.33 | 0.740 |
| UA (μmol/L), M (Q1, Q3) | 338.20 (254.35, 429.40) | 334.40 (247.15, 431.15) | 351.28 (268.98, 426.10) | Z=-0.84 | 0.399 |
| ALT (U/L), M (Q1, Q3) | 20.00 (14.00, 37.00) | 20.00 (14.00, 36.00) | 23.00 (14.75, 40.75) | Z=-0.97 | 0.332 |
| AST (U/L), M (Q1, Q3) | 53.00 (38.00, 80.50) | 54.00 (39.00, 79.00) | 50.00 (35.50, 103.50) | Z=-0.60 | 0.546 |
| ALB (g/L), M (Q1, Q3) | 31.40 (27.80, 35.05) | 31.50 (28.20, 35.00) | 30.95 (27.30, 35.15) | Z=-1.02 | 0.307 |
| FBG (g/L), M (Q1, Q3) | 2.77 (2.04, 3.49) | 2.77 (2.07, 3.44) | 2.75 (1.80, 3.53) | Z=-0.54 | 0.590 |
| INR, M (Q1, Q3) | 1.18 (1.09, 1.31) | 1.18 (1.09, 1.31) | 1.17 (1.09, 1.28) | Z=-0.13 | 0.894 |
| PCO₂ (mmHg), M (Q1, Q3) | 33.40 (30.40, 36.75) | 33.20 (30.30, 36.85) | 34.00 (31.28, 36.20) | Z=-0.95 | 0.343 |
| SBC (mmol/L), M (Q1, Q3) | 23.59 (22.40, 24.70) | 23.59 (22.40, 24.75) | 23.65 (22.40, 24.50) | Z=-0.05 | 0.957 |
| Ejection fraction (%), M (Q1, Q3) | 61.76 (60.00, 65.00) | 61.76 (60.00, 65.00) | 61.76 (60.00, 65.00) | Z=-0.14 | 0.887 |
| ***C. Intraoperative variables*** | | | | | |
| Extracorporeal circulation time (min), M (Q1, Q3) | 205.00 (175.00, 234.50) | 205.00 (175.00, 235.00) | 205.00 (175.75, 234.00) | Z=-0.14 | 0.891 |
| Aortic cross-clamp time (min), M (Q1, Q3) | 114.00 (94.00, 136.00) | 113.00 (93.00, 135.00) | 116.50 (94.75, 142.25) | Z=-0.82 | 0.413 |
| Surgical time (h), M (Q1, Q3) | 7.94 (7.00, 8.50) | 7.94 (7.00, 8.50) | 7.94 (7.00, 8.52) | Z=-0.35 | 0.727 |
| Isolated ascending aortic replacement (AAR), n (%) | 135 (22.54) | 115 (24.01) | 20 (16.67) | χ²=2.96 | 0.085 |
| Aortic root replacement, n (%) | 249 (41.57) | 194 (40.50) | 55 (45.83) | χ²=1.12 | 0.289 |
| Wheat's procedure, n (%) | 2 (0.33) | 2 (0.42) | 0 (0.00) | - | 1.000 |
| DAVID procedure, n (%) | 1 (0.17) | 1 (0.21) | 0 (0.00) | - | 1.000 |
| Partial arch replacement, n (%) | 35 (5.84) | 27 (5.64) | 8 (6.67) | χ²=0.19 | 0.667 |
| Total arch replacement with FET (TAR-FET), n (%) | 430 (71.79) | 337 (70.35) | 93 (77.50) | χ²=2.42 | 0.120 |
| CABG, n (%) | 38 (6.34) | 30 (6.26) | 8 (6.67) | χ²=0.03 | 0.871 |
| *Footnote. t: t-test; Z: Mann-Whitney test; χ²: Chi-square test; -: Fisher's exact test. SD: standard deviation; M: median; Q1: 1st quartile; Q3: 3rd quartile. Surgical-strategy variables (AAR, aortic root replacement, partial arch replacement, TAR-FET) are not mutually exclusive; every patient underwent some form of ascending-aortic intervention. Preoperative CRP was excluded due to >50% missing values.* | | | | | |

**Supplementary Table 2. Sensitivity analysis: comparison of the final nomogram and a re-fitted model without serum creatinine.**

| **Model variant** | **Variables included** | **Training AUC (95% CI)** | **Validation AUC (95% CI)** |
| --- | --- | --- | --- |
| Final nomogram (in main text) | 10 variables (incl. Cr) | 0.796 (0.756–0.835) | 0.765 (0.678–0.852) |
| Sensitivity model: Cr removed | 9 variables (excl. Cr) | 0.791 (0.751–0.831) | 0.762 (0.672–0.851) |
| *Footnote. Re-fitted multivariable logistic regression after omitting serum creatinine. DeLong test for paired AUCs: training P = 0.225, validation P = 0.639 (no statistically significant difference between models). Brier scores: training 0.182 (10-var) vs 0.185 (9-var); validation 0.187 vs 0.188. Omitting Cr forced compensatory inflation of the UA coefficient (per-μmol/L OR rose from 1.0022 to 1.0035, P < 0.001), the statistical signature of two collinear renal-function variables. The 10-variable model was therefore retained as the primary nomogram. AUC: area under the receiver operating characteristic curve; CI: confidence interval; Cr: serum creatinine; UA: uric acid.* | | | |

**Supplementary Table 3. Performance of the nomogram at the optimal Youden-index cut-off in both cohorts.**

| **Cohort** | **AUC (95% CI)** | **Cut-off** | **Sensitivity** | **Specificity** | **PPV** | **NPV** |
| --- | --- | --- | --- | --- | --- | --- |
| Training (n = 479) | 0.796 (0.756–0.835) | 0.484 | 78.6% | 66.7% | 74.6% | 71.4% |
| Validation (n = 120) | 0.765 (0.678–0.852) | 0.536 | 76.0% | 64.4% | 78.1% | 61.7% |
| *Footnote. Cut-off values were determined by the Youden index on each cohort. AUC: area under the receiver operating characteristic curve; CI: confidence interval; PPV: positive predictive value; NPV: negative predictive value.* | | | | | | |

**Supplementary Table 4. CRP multiple-imputation sensitivity analysis.**

*Re-fitting the model after multiple imputation of preoperative CRP did not improve discrimination and the pooled CRP coefficient was not statistically significant.*

| **Analysis** | **AUC (training)** | **Pooled OR for CRP (95% CI)** | ***Pooled P*** |
| --- | --- | --- | --- |
| Primary 10-variable model (CRP excluded) | 0.7959 | — (not in model) | — |
| 11-variable model with multiply-imputed CRP | 0.7971 (mean, SD 0.0012) | 1.003 (0.981–1.026) | 0.79 |
| *Footnote. Multiple imputation by chained equations (iterative imputer with Bayesian-ridge predictor, m = 20 imputations, posterior sampling on). Estimates pooled using Rubin's rules. The pooled OR for CRP is non-significant and ΔAUC over the primary model is +0.001, confirming that CRP does not contribute meaningful information beyond the other 10 predictors. CRP: C-reactive protein; OR: odds ratio; CI: confidence interval; AUC: area under the receiver operating characteristic curve.* | | | |
